# Autoimmune encephalitis in humans: how closely does it reflect multiple sclerosis ?

**DOI:** 10.1186/s40478-015-0260-9

**Published:** 2015-12-04

**Authors:** Romana Höftberger, Marianne Leisser, Jan Bauer, Hans Lassmann

**Affiliations:** Institute of Neurology, Medical University of Vienna, Vienna, Austria; Center for Brain Research, Medical University of Vienna, Vienna, Austria

**Keywords:** Multiple sclerosis, Experimental autoimmune encephalomyelitis, Inflammation, Demyelination, Neurodegeneration

## Abstract

**Introduction:**

Multiple sclerosis (MS) is a chronic inflammatory demyelinating disease of the central nervous system. Immunological studies suggest that it is a T-cell mediated autoimmune disease, although an MS-specific target antigen for autoimmunity has so far not been identified. Models of experimental autoimmune encephalomyelitis in part reproduce features of MS, but none of the models so far covers the entire spectrum of pathology and immunology. Autoimmune disease of the nervous system has occasionally been observed in humans after active sensitization with brain tissue or brain cells, giving rise to acute demyelinating polyradiculoneuritis, acute disseminated encephalomyelitis and in rare cases reflecting an inflammatory demyelinating condition similar to acute multiple sclerosis. In this study we analyzed in detail the immunopathology in archival autopsy tissue of a patient who died with an MS like disease after repeated exposure to subcutaneous injections of lyophilized brain cells.

**Results:**

The pathology of this patient fulfilled all pathological diagnostic criteria of MS. Demyelination and tissue injury was associated with antibody (IgM) deposition at active lesion sites and complement activation. Major differences to classical EAE models were seen in the composition of inflammatory infiltrates, being dominated by B-cells, infiltration of IgM positive plasma cells, profound infiltration of the tissue by CD8^+^ T-lymphocytes and a nearly complete absence of CD4^+^ T-cells.

**Conclusions:**

Our study shows that auto-sensitization of humans with brain tissue can induce a disease, which closely reflects the pathology of MS, but that the mechanisms leading to demyelination and tissue injury differ from those, generally implicated in the pathophysiology of MS through studies in experimental autoimmune encephalomyelitis.

## Introduction

Multiple sclerosis (MS) is a chronic inflammatory disease of the central nervous system, which leads to plaque like primary demyelination in the white and grey matter and focal as well as diffuse neurodegeneration [33]. Immunological studies suggest that autoimmunity against nervous system antigens plays a major role in its pathogenesis, although so far no MS-specific autoimmune reaction has been identified [24]. Auto-sensitization of rodents and primates with brain tissue, myelin or with brain proteins, induces experimental autoimmune encephalomyelitis (EAE), which is generally regarded as the most suitable animal model for MS. [18] However, EAE pathology only incompletely mimics that seen in MS, and it is currently unclear, whether the differences are due to the genetic background or the environmental exposure between animals and humans or whether there are more fundamental differences in pathogenesis. A detailed analysis of the immunopathology of autoimmune encephalomyelitis in humans could fill this gap of knowledge.

Autoimmune disease in humans after active sensitization with brain tissue is a rare event. In a detailed epidemiological meta-analysis, based on more than 100.000 individuals exposed to brain tissue containing rabies vaccine, an incidence of neuroparalytic autoimmune complications has been described as 0.3 to 1 out of 1000. The incidence was higher in adults compared to children and individuals with Caucasian genetic background were more frequently affected compared to others. Furthermore, the incidence was dependent upon the preparation of the vaccine [50]. Similar autoimmune complications have been observed, following treatment of patients with fresh or lyophilized brain cells, which was used in alternative medicine in the first half of the XX^th^ century [7, 46]. Most recently, neurological complications have been described in workers of a slaughter house, who were chronically exposed to an aerosole of brain tissue at their working place [31]. Inflammatory polyradiculoneurits was seen in 60 to 70 % of patients, while the rest presented with a disease affecting the central nervous system, mainly reflecting acute disseminated encephalomyelitis or transverse myelitis [50]. Considering the similarity of these diseases with autoimmune encephalomyelitis or neuritis in rodents, it is likely that these disorders are mediated by CD4^+^ autoreactive T-cells [2]. In rare cases, however, an inflammatory demyelinating phenotype was observed resembling acute MS. [46, 54] A detailed pathological analysis of such cases with inflammatory demyelinating disease, using new technology, which have been developed for the immunopathological characterization of MS lesions, could provide answers, to what extent the spectrum of brain alterations in MS patients may be reproduced in a defined human brain autoimmune disease and about immune mechanisms, which are associated with the demyelinating disease phenotype.

In this study we retrieved one of these cases from the pathology archive of the Institute of Neurology of the Medical University of Vienna ([46]; the only case for which archival pathological material was still available after international search) and compared its pathology in detail with that, defined in recent years in MS patients [33, 36]. In addition, we compared the changes in this case with those seen in EAE in primates (marmosets, [26, 27]) and in the commonly used chronic mouse model, induced by sensitization of C57B6 mice by active sensitization with myelin oligodendrocyte glycoprotein [18, 44]. The study was approved by the local Ethical Commission of the Medical University of Vienna (EK. Nr. 097/01/2015).

## Material and methods

Detailed clinical information of the disease course of the patient with demyelinating human autoimmune encephalitis (HAE) has been provided before [46]. In short a 51-year-old male patient presented with a mild slowly progressive hemi-Parkinson syndrome, starting 4 years before his death. Due to the lack of other therapeutic options the patient was treated over a period of 17 months with 7 injections of lyophilized calf brain cells (derived from cortex, thalamus, hypothalamus, and striatum) and placenta cells (0.02 g dry weight in 6 to 8 ml of physiological saline) at two to four month intervals. The first 6 injections were well tolerated. Twenty two days after the seventh injection the patient developed progressive right-sided hemiparesis. This was followed by rapid deterioration of the neurological status, leading to paraparesis, spasticity of the lower extremities, positive Babinski reflex, and tremor, and finally progressed to a comatous state. Seven weeks after onset of the neurological disease the patient died due to cardio-respiratory failure. Brain autopsy revealed large bilateral periventricular lesions together with multiple small focal peri-venous lesions in the cerebral white matter. Histological analysis showed active inflammatory demyelinating lesions in the cerebral white matter and some small inflammatory lesions in the cerebellum, brain stem, and spinal cord. In addition, neuronal loss and the presence of some neurons with Lewy body inclusions were seen in the right substantia nigra [46].

The comparison is based on archival autopsy material of MS cases (including acute MS, relapsing MS and primary or secondary progressive MS; [14, 17]), of brain samples of EAE in marmosets (induced by active sensitization with recombinant MOG_;_ [26, 27]) and of chronic mouse EAE (induced by active sensitization with MOG_35–55;_ [18, 44]), all contained in the archive of the Center for Brain Research.

### Pathological analysis

**Fig. 1:**
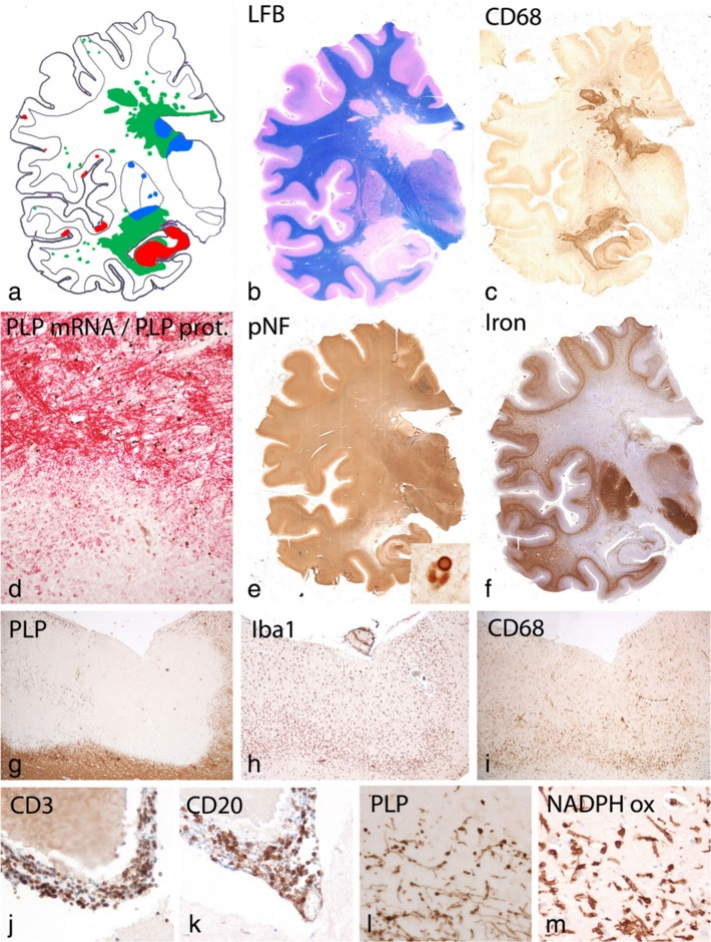
Basic Pathology of HAE: **a**: Topographical distribution of demyelinated lesions in the brain shows the prominent periventricular demyelination with peri-venous extensions (Dawson Fingers) and demyelinated plaques in the cortex and the deep grey matter nuclei; green: white matter lesions, red: cortical and hippocampal lesions, blue: lesions in thalamus and basal ganglia; the blue dots in the meninges show the location of prominent meningeal inflammatory infiltrates; **b**: Sections stained with luxol fast blue depicts the demyelinated lesions in the white matter; **c**: Immunocytochemistry for CD68 shows the accumulation of macrophages at the edge of active white matter lesions; **d**: Double staining for proteolipid (PLP) protein (red) and mRNA (black) reveals loss of oligodendrocytes within the lesion and the presence of numerous macrophages with PLP degradation products at the lesion edge; **e**: In sections stained for phosphorylated neurofilament only a mild to moderate reduction of axonal density is seen in the lesions; the insert shows a neuron in the substantia nigra with an α-synuclein reactive Lewy body. **f**: The section stained for iron shows prominent iron accumulation in the deep grey matter nuclei and at the cortico/subcortical border; some increased iron reactivity is seen within the periventricular demyelinated lesions: **g**: Subpial cortical lesion in the insular cortex (Fig. 1**a**) with selective loss of myelin in the cortex; **h**, **i**: The subpial lesions shows an actively demyelinating edge with high density of activated microglia (Iba-1, Fig. 1**h**), expressing the phagocytosis associated marker CD68 (Fig. 1**i**). **j**, **k**: In the meninges, covering the active lesion, inflammatory infiltrates are seen, composed or perivascular T-cells (CD3, Fig. 1**j**) and B-cells (CD20, Fig. 1**k**); **l**: The active lesion edge of the cortical lesions contains numerous macrophages with PLP reactive myelin degradation products; **m**: Activated microglia and macrophages at the lesions edge express NADPH oxidase

For detailed pathological analysis of the HAE case, one hemispheric paraffin embedded block through the mid thalamus was available (Fig. 1). Hemispheric sections were stained with hematoxylin eosin, Luxol fast blue myelin stain, Bielschowsky silver impregnation for axons, and with the modified Turnbull reaction for iron. Immunocytochemistry was in part performed on the entire hemispheric section or, alternatively, in dissected hemispheric sections, containing the lesions, the normal appearing white matter and the cerebral cortex.

Immunocytochemistry was performed on de-paraffinized sections using the primary antibodies listed in Table 1. This table also contains information on methods of antigen retrieval. Visualization of bound primary antibodies was done with a biotin avidin technique ([13], Table 1). In situ hybridization for proteolipid protein mRNA was performed according to Breitschopf et al. [8] and TUNEL staining according to Gold et al. [19]. To determine the proliferation rate of T-cells and B-cells, the sections were first stained for proliferating cell nuclear antigen (PCNA) using an alkaline phosphatase technique followed by the respective markers CD3 and CD20, visualized with a biotin-avidin-peroxidase method. In situ hybridization for Epstein Barr Virus (EBER) was performed using the EBER pNA detection kit (DAKO Y5200), including an EBV^+^ cerebral lymphoma as a positive control.

**Table 1:** Antibodies used for immunocytochemistry

Primary antibody	Antibody type	Target	Source	Staining
PLP	Mouse (mAB)	Proteolipid protein	MCA8394; AbD Serotec	1:1000; E
MAG	Mouse (mAB)	Myelin-associated glycoprotein	ab89780; Abcam	1:1000; E
MOG	Mouse /mAB)	Myelin oligodendrocyte glycoprotein	8-18C5; C. Lininton, Cardiff, UK	1:1000; C
GFAP	Mouse (mAB)	Glial fibrillary acid protein	0410080; ThermoSc	1:200; E
pNF	Mouse (mAB)	Phosphorylated Neurofilament	Affinity, SMI31,NA1219, Exeter, UK	1:20000; E
APP	Mouse (mAB)	Amyloid precursor protein	MAB348; Chemicon, Temecula, CA, USA	1:1000; C
Iba1	Rabbit (pAB)	Ionized calcium binding adaptor molecule 1	019-19741; WAKO Chemicals, Neuss, Germany	1:3000; E
CD68	Mouse (mAB)	Cluster of Differentiation 68	M0814; Dako	1:100; E
p22phox	Rabbit (pAB)	NADPH oxidase	Sc-20781; Santa Cruz,	1:100; C
CD3	Rabbit (mAB)	T-cells	RM-9107-S; Neomarkers, Fremont, CA, USA	1:2000; E
CD4	Mouse (mAB)	CD4 T-cells	ACRIS, 1F6; DM-119-05, San Diego, CA, USA	1:1000; E
CD8	Mouse (mAB)	CD8 T-cells	M7103; Dako, Glostrup, Denmark	1:250; E
CD20	Mouse (mAB)	B-cells	MS-340-S; Neomarkers, Fremont, CA, USA	1:100; E
CD138	Mouse (nAB)	Plasma Cells	Serotec, MCA 681H; UK	1:500; E
GranB	Mouse (mAB)	Granzyme B	MS-1157-S; Neomarkers, Fremont, CA, USA	1:1000; E
IgG	Rabbit (pAB)	Imunoglobulin G	A0423, DAKO, Glostrup, DK	1:400; Prot.
IgA	Rabbit (pAB)	Immunoglobulin A	A042, DAKO, Glostrup, DK	1:2000; Prot.
IgM	Rabbit (pAB)	Immunoglobulin M	A426; DAKO, Glostrup, DK	1:400; Prot
C9neo	Rabbit (pAB)	Compement C9neo antigen	P. Morgan; Cardiff, UK	1:2000; Prot
PCNA	Mouse (mAB)	Proliferating cell nuclear antigen	M0879, DAKO, Glostrup, DK	1:10000; C
E06	Mouse (mAB)	Oxidized phospholipids	Avantilipids; 330001S, Alabaster, AL, USA	10μg/ml; none
α-Syn	Mouse (mAB)	α-Synuclein	Aj.ROBOSCREEN, mab 5G4	1:1000; C
AT8	Mouse (mAB)	Phosphorylated tau-protein (PHF)	Immunogenetics, Ghent, Belgium	1:2000; C
AIF	Rabbit (pAB)	Apoptosis inducing factor	Milipore, AB16501; Temecula, CA	1:250; C

*mAB* monoclonal antibody, *pAB* polyclonal antibody, *C* citrate buffer pH 6, *E* ethylenediaminetetraacetic acid buffer pH 9.0, *prot* Protease XXIV; 0.03 %, 15 min

Quantitative analysis of axonal density was performed in the microscope at a magnification of 20x in sections stained for phosphorylated neurofilaments. The number of axons, crossing a line of 2 mm were determined by manual counting at three sites of the lesions and the normal appearing white matter. A similar approach was also used to determine the number of axons with disturbance of axonal transport, reflected by the positivity for amyloid precursor protein, and for the presence of axons with terminal end bulbs.

## Results

### Basic pathology of human autoimmune encephalitis (HAE)

**Fig. 2:**
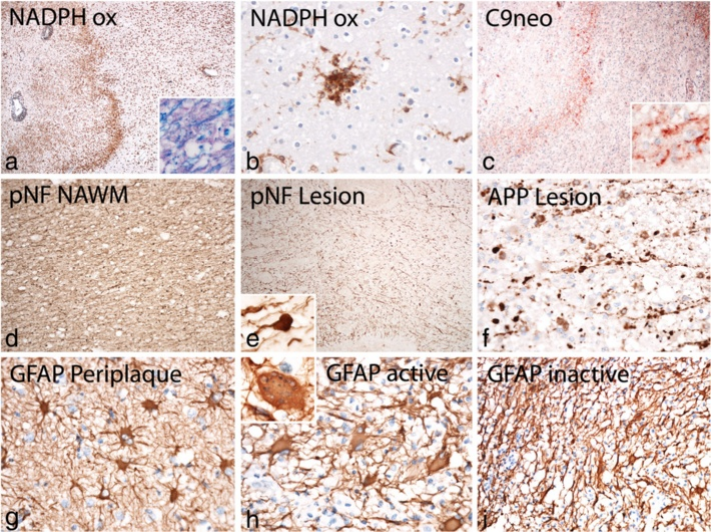
Demyelination and gliosis in active white matter lesions: **a**: Activity at the edge of the lesion is associated with densely packed activated macrophages and microglia, expressing NADPH oxidase; profound activation of microglia is also seen in the peri-plaque white matter; the insert show the presence of luxol fast blue positive myelin degradation products within macrophages; **b**: In the peri-plaque white matter numerous microglia nodules are present, expressing NADPH oxidase; **c**: At the active lesion edge profound deposition of activated complement (C9neo antigen) is visible, which appears to dress degenerating myelin (insert); **d**, **e**: Staining for phosphorylated neurofilament shows reduction of axonal density within the lesion (**e**) in comparison to the peri-plaque white matter (**d**); axonal spheroids or end bulbs are mainly present at the lesion edge (e, insert); **f**: dystrophic axons at the lesion edge show accumulation of amyloid precursor protein (APP) as a sign of disturbed axonal transport; **g**, **h**: Profound protoplasmic astrocytic gliosis is seen in sections stained for glial fibrillary acidic protein (GFAP); some of the astrocytes resemble the pathological changes of Creutzfeldt Peters cells (insert in h). **i**: Fibrillary astrocytic gliosis is seen in the demyelinated lesion center

**Fig. 3:**
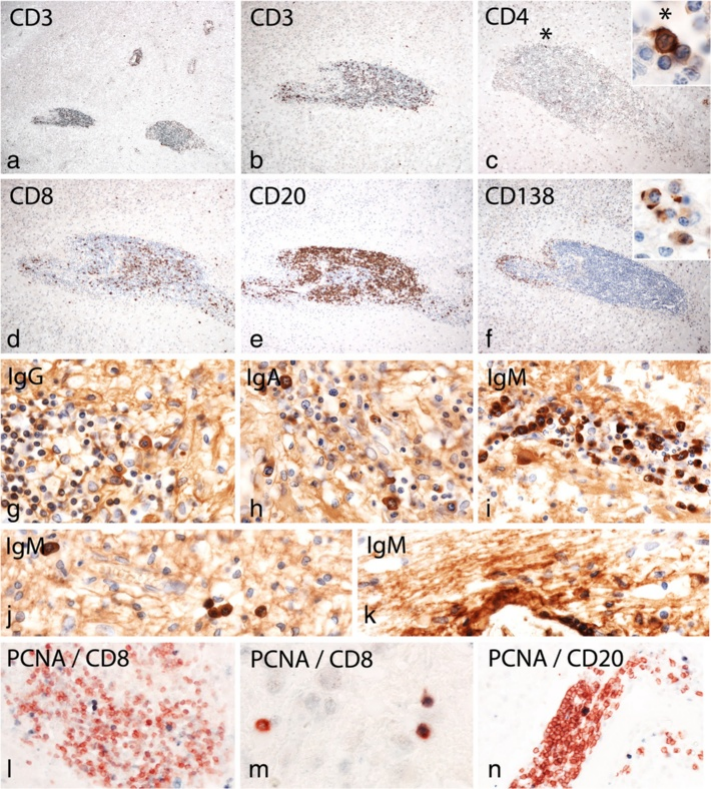
Patterns of inflammation in HAE: **a**: Within the demyelinating lesions a very pronounced inflammatory reaction is seen, characterized by the presence of numerous densely packed perivascular inflammatory cuffs; **b**, **c**, **d**: The inflammatory cuffs contain high numbers of CD3^+^ and CD8^+^, but only exceptionally CD4^+^ T-cells; the insert in c shows the single CD4^+^ T-cells within the entire inflammatory cuff at high magnification; CD3^+^ and CD8^+^ T-cells are also seen diffusely infiltrating the lesion parenchym (d, d). **e**: CD20^+^ B-cells are the dominant leukocyte population within the perivascular cuffs; **f**: In addition to T-cells and B-cells the inflammatory infiltrates also contain CD138^+^ plasma cells, shown at high magnification in the insert; note that the T-cells, B-cells and plasma cells are located in different sub-regions of the cuff, similar to that seen in lymph follicle like structures, present in chronic inflammatory conditions; **g**-**i**: The majority of plasma cells within the infiltrates contain IgM (**i**), while the number of IgG (**g**) and IgA (**h**) positive plasma cells is low; **j**: IgM containing plasma cells are also diffusely dispersed in the lesion parenchyma; k: at the lesion edge IgM is seen associated with myelinated fibers; l-n: Double staining of sections with the proliferation marker PCNA and leukocyte markers shows local proliferation of CD8^+^ T-cells and CD20^+^ B-cells in perivascular cuffs, while in the parenchyma of the lesion only proliferating CD8^+^ T-cells are seen

Analysis of the entire brain hemispheric sections showed large periventricular inflammatory demyelinating lesions with partial preservation of axons (Fig. 1). Peri-venous finger like extensions of the lesions into the adjacent white matter were prominent (Dawson Fingers, [11]). In addition, numerous small peri-venous areas of demyelination were seen in the entire cerebral white matter. Demyelination was also present in the cortex and in deep grey matter nuclei as previously described in MS. [30] In the cortex small intra-cortical and larger subpial lesions were visible. Grey matter demyelination was particularly pronounced in the hippocampus (Fig. 1a and b). The vast majority of the lesions showed active demyelination at the edges, characterized by a dense rim of activated macrophages and the presence of early myelin degradation products within macrophages and microglia Fig. 1c, d, Fig. 2a). Demyelination was associated with complete loss of oligodendrocytes (Fig. 1d) and remyelination was absent. TUNEL staining revealed some cells with DNA fragmentation with a morphological appearance of necrosis at the edge and in the center of the lesions. Nuclear accumulation of apoptosis inducing factor, a hallmark of cell degeneration in Pattern III lesions of multiple sclerosis [56] was not found. Profound microglia activation was also seen in the peri-plaque white matter (Fig. 2a) and clusters of activated microglia (so called microglia nodules, [41]) were abundant (Fig. 2b). Staining for neurofilament revealed only a moderate reduction of axons within the demyelinated lesions (Figs. 1e, 2e) in comparison to the adjacent normal appearing white matter (Fig. 2d). The reduction of axonal density was 23 % at the lesion edges and 34 % in the lesion center. However, axonal spheroids and end bulbs (Fig. 2e) reactive for amyloid precursor protein (APP, [13]) were numerous in particular at the active edge of the lesions (Fig. 2f). At the lesion edge 32 % of the axons showed accumulation of APP, but only 12 % revealed the presence of axonal end bulbs as an indicator for axonal transection. Highly activated “protoplasmic” astrocytes were dispersed at sites of active demyelination and in the peri-plaque white matter (Fig. 2g,h), and some of them contained multiple nuclei or nuclear fragments (Creutzfeldt Peters cells; Fig. 2h insert). Fibrillary astrocytic gliosis was seen in the inactive lesion center (Fig. 2i). Active demyelination was associated with deposition of activated complement (C9neo antigen; Fig. 2c) in all lesions with initial stages of myelin destruction. This was associated with dressing of myelinated fibers by immunoglobulin (IgM; Fig. 3l).

Actively demyelinating lesions were also seen in the cortex (Fig. 1a). Fig. 1g shows an active subpial lesion in the insular cortex. Active myelin destruction was associated with activated microglia (Fig. 1h), containing myelin degradation products (Fig. 1l) and expressing the phagocytosis associated marker CD68 (Fig. 1i) and NADPH oxidase (Fig. 1m). Active cortical demyelination was topographically related to meningeal inflammation, consistent of CD3 positive T-cells (Fig. 1j) and CD20 positive B-cells (Fig. 1k). However, no deposition of activated complement was seen in active cortical lesions at the site of active demyelination, as described before in MS. [9].

Overall these data show, that the lesions in HAE reflect all pathological hallmarks seen in classical MS, following a pattern of demyelination, which is associated with antibody and complement deposition (Pattern II, [36]). Pattern III demyelination, defined by loss of myelin associated glycoprotein, distal oligodendrogliopathy, oligodendrocyte apoptosis and a concentric type of demyelination [1] were not encountered in this patient.

### Inflammatory reaction

The demyelinating pathology, described above occurred on a background of a very severe inflammatory reaction (Fig. 3). Numerous very large inflammatory infiltrates were present within the lesions, while inflammation was sparse in the normal appearing white matter and virtually absent in the cortex. However, inflammation was present in the leptomeninges in close proximity to cortical demyelinated lesions (Fig. 1a, j, k). The inflammatory infiltrates contained high numbers of CD3^+^ T-cells (Fig. 3 b) and a very similar number of CD8^+^ cells (Fig. 3d). In contrast, CD4^+^ T-cells were exceptionally rare. As an example, in the very large inflammatory infiltrate, depicted in Fig. 3c, only one single CD4^+^ T-cell was found (asterisk and insert). By far, however, the most numerous components of the inflammatory infiltrates were CD20^+^ B-cells, which outnumbered T-cells by a factor of 3 to 4 (Fig. 3e). In addition, CD138 positive plasma cells were seen in separated regions of the infiltrates (Fig. 3f), which mainly contained IgM (Fig. 3i) but only occasionally IgG (Fig. 3g) or IgA (Fig. 3h). The large inflammatory infiltrates in the perivascular spaces and meninges showed clear separation of T-cell, B-cell and plasma cell dominated regions in a pattern, similar to that described in lymph-follicle like inflammatory aggregates in MS. [47] Diffuse inflammatory infiltrates within the lesion parenchyma mainly consisted of CD8^+^ T-cells (Fig. 3d) and IgM^+^ plasma cells (Fig. 3j). At the lesions edges, dressing of myelin sheaths with IgM was noted (Fig. 3k). Proliferation of leukocytes was determined by the expression of the proliferation marker PCNA in T-cells and B-cells. Double stained cells were CD8^+^ cells in perivascular cuffs and in diffuse infiltrates in the parenchyma (Fig. 3l, m) and CD20^+^ perivascular cells (Fig. 3n). Quantitative data on the percentage of different leukocyte subsets in perivascular cuffs are provided in Table 2.

**Table 2:** Percentage of specific leulocyte subsets of total perivascular cells in inflammatory cuffs in HAE

Leukocyte	Percentage of Total Cells / Cuff
CD3	21
CD4	<0.1
CD8	20
CD20	72
CD138	9
IgM	7
IgG	2
IgA	0.7
% proliferating CD8^+^ cells	2.3
% proliferating CD20^+^ cells	3.5

Counts are based on a total of 400 cells / cuff / marker; for enumerating CD4^+^ T-cells a total of 3.000 cells / cuff were analysed

**Fig. 4:**
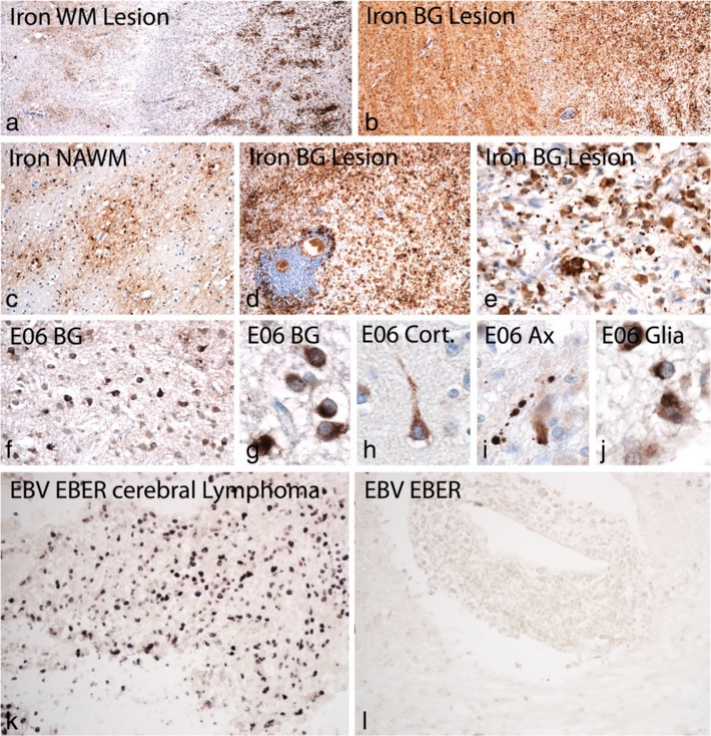
Iron deposition and oxidative injury in HAE: **a**: Related to white matter lesions a moderate iron deposition is seen in the peri-plaque white matter (left side), while very little iron is present in the active edge of the lesion; in the lesion center numerous iron containing macrophages are present (right side); **b**: In contrast, in lesions in the basal ganglia massive iron deposition is present in the peri-plaque grey matter and a high content of iron containing macrophages and microglia (left side) are seen within the lesion (right side); **c**: Higher magnification shows the presence of iron in oligodendrocytes and myelin in the normal appearing white matter; **d**, **e**: in basal ganglia lesions iron is mainly seen in microglia and macrophages (**d**), but is also present in the form of small extracellular iron granules (**e**); **f**-**j**: Oxidative injury is visualized by the cytoplasmic accumulation of oxidized phospholipids (E06 reactivity); it is mainly seen in basal ganglia lesions in neurons (**f**, **g**) in dystrophic axons (**i**) and in astrocytes and oligodendrocytes (**j**); as described before in MS lesions E06 reactivity is present diffusely within the cytoplasm of neurons and oligodendrocytes, while in astrocytes (**j**) it is sequestered in cytoplasmic granules, possibly reflecting autophagy of damaged cell components; only very few cortical neurons show cytoplasmic accumulation of oxidized phospholipids at sites of lesion activity (**h**). **k**, **l**: Cells with nuclear EBV / EBER reactivity are present in high numbers in a control case of EBV positive cerebral lymphoma (**k**), while no single cell with EBER reactivity is seen in the inflammatory cuffs or the lesion parenchyma in the HAE case

The unusually high B-cell component within the lesions may indicate Epstein Barr Virus infection of these cells, as suggested before to occur in MS lesions [48]. To address this question we performed in situ hybridization for EBV/EBER using an autopsy case with a cerebral EBV related lymphoma as a positive control, which showed prominent EBV EBER signal in the nucleus of malignant B-cells (Fig. 4k). In contrast, no single EBER positive cell was found in the HAE case (Fig. 4l). It is unlikely that the absence of EBER reactivity within our case of HAE is due to technical reasons, since EBER in situ hybridization provides consistent results in human autopsy brain tissue [32] and the quality of tissue preservation, including mRNA preservation, in our case of HAE was better compared to that seen in other routine autopsy tissues (see in situ hybridization for proteolipid protein mRNA shown above).

### Oxidative injury in neurons and glia

Oxidative injury has recently been identified as a major tissue alteration associated with demyelination and neurodegeneration in MS lesions [14] and it was suggested that oxidative injury may be amplified in the presence of iron in the aging human brain and its liberation in active MS lesions [22]. In line with the age of the patient profound iron accumulation was found in the brain (Fig. 1f), which was most prominent in the basal ganglia and the substantia nigra, followed by the cortico/subcortical border zone. Iron reactivity was mainly seen in myelin and oligodendrocytes as described before ([20]; Fig. 4a, c). Active areas of the white matter lesions contained a low iron load, but iron accumulated in macrophages in the lesion center (Fig. 4a). In contrast, massive iron load was present in lesions, which affected the basal ganglia (Fig. 4b). As described before in MS, iron was lost from myelin and oligodendrocytes and accumulated in macrophages and microglia in these lesions (Fig. 4d). In addition, granular iron reactivity was present in the extracellular space, as described before in MS lesions [22]. Oxidative injury, reflected by the cytoplasmic accumulation of oxidized lipids in white matter lesions and the cortex was low in comparison to that seen in acute MS cases with Pattern III pathology or in the lesions of patients with progressive MS. [14, 21] However, in the lesions affecting areas with high iron content in the basal ganglia accumulation of oxidized phospholipids was seen in neurons, dystrophic axons and glia (Fig. 4f-j).

### Pathological changes in the substantia nigra

Consistent with the clinical presentation, moderate changes of Parkinson’s disease were seen in the substantia nigra, characterized by a mild reduction of pigmented cells and the presence of few cells with Lewy bodies, reactive for α-synuclein (Fig. 1e, insert). No Lewy bodies were seen in the hippocampus or cortex (Braak&Braak stage III of Parkinson´s disease; brainstem predominant Lewy body type pathology according to McKeith criteria). Despite the presence of neurodegeneration the substantia nigra was not targeted by the inflammatory or demyelinating process.

### Comparison of HAE with MS and EAE in rodents and primates

Similarities and differences between the case of HAE and MS or animals models of EAE are summarized in Table 3. Most pathological hallmarks, considered to be specific for MS, were shared between HAE and MS. One major difference was that none of the lesions showed a demyelinating pattern, characterized by distal oligodendrogliopathy and oligodendroglia apoptosis (pattern III; [34]). This pattern of demyelination seems to represent an extreme example of tissue injury mediated by oxidative and mitochondrial injury [14, 38] and its absence in this case is in line with the relatively mild oxidative injury, which was largely restricted to areas with high iron content and liberation into the lesions.

A major quantitative difference was also seen in the composition of inflammatory infiltrates. In the case described here the infiltrates largely contained CD20^+^ B-cells and CD8^+^ T-cells, while CD4^+^ lymphocytes were virtually absent. In addition plasma cells were found, which dominantly contained IgM. In MS inflammation is dominated by CD8^+^ T-cells and the number of CD20^+^ B-cells is much lower [16]. Furthermore, plasma cells in MS lesions mainly produce IgG and in much lower incidence IgA and IgM [12]. Also in MS CD4^+^ T-cells are sparse.

For the comparison with animal models we focused on the two extreme examples of the EAE spectrum, MOG peptide induced chronic EAE in C57Bl6 mice, which is a purely CD4^+^ T-cell mediated disease, which is most easy to induce and most reproducible, and is, thus, preferentially used in immunological studies of MS. [18] The other is the chronic EAE model induced by recombinant MOG in incomplete Freund’s adjuvant in marmoset monkeys, which appears to be most close regarding genetic background and method of sensitization to the case described in this study, but is heterogeneous between animals regarding clinical disease and severity of the pathological alterations [27]. The pathology of marmoset EAE revealed an inflammatory demyelinating disease in many essential criteria similar to MS and HAE, following a pattern of demyelination, that suggest involvement of demyelinating antibodies (Pattern II; [36]). There were, however some differences in astrocyte and microglia reaction and the inflammatory reaction in marmosets is dominated by T-cells, while the B-cell component is less pronounced and IgM producing plasma cells are sparse. Pathological alterations, related to iron deposition in the central nervous system and to oxidative injury were sparse or absent (Table 3).

**Table 3:** Comparison of HAE with MS and EAE Models

MS Pathology	AcuteMS	Progr. MS	Case HAE	Marmoset MOG EAE	Mouse MOG EAE
Demyelination					
DM Plaques	yes	yes	yes	yes	yes^a^
Perivenous / Confluent	yes	yes	yes	yes	yes
Periventricular	yes	yes	yes	yes	no
Slowly expanding lesions	no	yes	no	no	no
Dawson Fingers	yes	yes	yes	yes	no
Leukocortical Lesions	yes	yes	yes	yes	no
Intractortical Lesions	yes	yes	yes	yes	no
Subpial lesions	few	many	few	yes	no
Shadow plaques	few	var	no	yes	no
Pattern II DM					
C9neo Deposition	var	few	yes	yes	no
Ig Deposition	var	var	yes	yes	no
Macrophage association	yes	yes	yes	yes	yes
Pattern III DM					
Pre-phagocytic Lesions	var	few	no	no	no
Oligodendrocyte Apoptosis	var	var	no	no	no
Concentric DM Pattern	var	rare	no	no	no
Axonal / Neuronal Pathology					
Relative axonal pres.	yes	yes	yes	yes	no
Acute injury in act. lesions	yes	yes	yes	few	yes
Diffuse axonal Injury	few	mod	few	few	few
Retrograde Neurodegeneration	few	yes	few	n.d.	yes
Astrocyte Pathology					
Protoplasmic gliosis in active lesions	yes	yes	yes	mild	yes
Creutzfeldt Peters Cells	many	few	many	no	no
Fibrillary Gliosis in inactive lesions	yes	yes	yes	yes	yes
Aquaporin 4 loss	Var (Pat III)	no	no	n.d.	no
Inflammation					
CD3	many	many	many	many	many
CD4	few	few	single	n.d.	many
CD8	many	many	many	n.d.	few
CD20	mod	mod	massive	mod	few
Plasma Cells	mod	mod	mod	mod	few
Follicle like structures	var	var	yes	yes	no
IgG PC	many	many	few	mod	n.d.
IgA PC	few	few	few	n.d.	n.d.
IgM PC	few	few	many	few	n.d.
PCNA CD8	yes	yes	yes	n.d.	n.d.
PCNA CD20	yes	yes	yes	n.d.	n.d.
Microglia / Macrophages					
Iba1 (active lesions)	many	many	many	many	many
CD68 (active lesions)	many	many	many	n.d.	many
NADPH oxidase microglia	many	many	many	n.d.	no
Microglia nodules	many	mod	many	few	no
Diffuse activation NAWM/GM	mod	massive	mod	no	mod
Iron Related Pathology					
Iron accumulation in NAWM/GM	Age related	Age related	Profound (age)	no	no
Iron loss in lesions	yes	yes	yes	no	no
Extracellular iron	yes	yes	yes	no	no
Uptake in Macrophages / MG	yes	yes	yes	few	no
Iron loss in PPWM	yes	yes	no	no	no
Oxidative Injury					
Ox Lipids OG and Myelin	yes	yes	few (iron)	no	no
Ox lipids neurons	yes	yes	few (iron)	no	no
EBV					
EBER positive B-cells	no	no	no	n.d.	n.d.

*Acute MS* Marburg type of acute MS, *Progr. MS* primary or secondary progressive MS, *Case HAE* human autoimmune encephalitis, described in this study, *Marmoset MOG EAE* chronic EAE induced in marmosets by active sensitization with recombinant myelin oligodendrocyte glycoprotein in incomplete Freund’s adjuvant, *Mouse MOG EAE* chronic EAE in C57Bl6 mice induced by active sensitization with MOG_35–55_ peptide

Yes^a^: white matter lesions in chronic mouse EAE are mainly due to massive axonal destruction and secondary demyelination

*Mod* moderate, *mild* mild, *few(iron)* only few cells in lesions with high iron content

In contrast to EAE in humans and primates, the lesions in chronic mouse models after active sensitization with MOG_35–55_, are fundamentally different in many respects. Primary demyelination is sparse and larger focal lesions are mainly due to inflammatory neurodegeneration with secondary demyelination. Inflammation is dominated by CD4^+^ T-cells and the contribution of other leukocyte subsets is sparse. Furthermore, macrophage and microglia reaction are profoundly different between this model and the human condition [44]. Finally, the extensive oxidative injury seen in the brain of patients with progressive MS, which is associated with slow expansion of pre-existing lesions and diffuse damage of the grey and white matter was neither reproduced in marmoset or mouse EAE models, nor in our case of HAE.

However, comparing EAE models with MS one has to consider that numerous different EAE models have been described, and the pathological alterations in the brain and spinal cord cover a broad spectrum, which resides between the two extremes, elaborated above in this study [34, 40, 42, 49, 52].

## Discussion

We show here that the vast majority of putative disease specific pathological alterations of MS are well reproduced in a patient with brain autoimmune disease induced by active sensitization with brain cells. However, inflammatory demyelination, as shown in this case, is not always a feature of autoimmune-disease following sensitization with brain antigens. In contrast, acute demyelinating polyradiculoneuritis and acute disseminated encephalomyelitis are more common than MS like inflammatory demyelination [2, 50]. In our case we found no clinical evidence that the patient suffered from MS prior to sensitization with brain antigens. Furthermore, all lesions in this patient presented with a similar stage of activity with active demyelination at the edge and an inactive lesion center, which was still diffusely infiltrated with macrophages. No separate inactive or remyelinated lesions were present in the entire brain. However, one can argue that MS like pathology is only seen in patients, who have a similar genetic predisposition compared to MS patients [20, 25], or who have been exposed to similar environmental challenges. In this case auto-sensitization with brain tissue would provide a trigger of the disease in a susceptible individual. As an example, EBV infection is associated with MS in epidemiological studies [3]. Presence of EBV infected B-cells in the MS brain has been described [48], but this observation could not be confirmed in other studies [32, 39, 58]. Despite the very high number of B-cells within the lesions in our case, we did not find a single EBV / EBER positive cell. If EBV exposure may have been involved in the development of the disease, it would have been in the peripheral immune system and not in the brain.

Alternatively, the mode of sensitization may have played a role in the induction of the demyelinating disease phenotype. A common feature of all patients, who developed HAE with widespread MS like primary demyelination was the immunization with native or phenol treated brain tissue in saline in the absence of adjuvants [28], which was applied by multiple injections over a prolonged time period [46, 54]. This was reproduced experimentally with a similar sensitization protocol in the earliest studies on EAE in monkeys [42]. With the introduction of Freund’s adjuvant in the immunization process the development of brain autoimmune disease was enhanced, resulting in reproducible disease models [29] by forcing mainly CD4^+^ T-cell driven inflammation [6]. Thus, the difference in the inflammatory response and the extent of demyelination between HAE, as shown in our current study, and classical EAE models may in part be related to the lack of immune stimulation by Freund’s adjuvant.

The nearly complete absence of CD4^+^ T-cells in the inflammatory infiltrates in the case described here indicates that other inflammatory cells, such as CD8^+^ T-cells or B-lymphocytes are important in driving inflammation and tissue injury in demyelinating HAE, while a disease phenotype of ADEM or polyradiculoneuritis may mainly be driven by a (CD4^+^) T-cell response, as suggested by its similarity to classical EAE models [2, 50]. However, ADEM pathogenesis may be heterogeneous, including also cases with additional presence of potentially demyelinating anti-MOG antibodies [57]. In multiple sclerosis lesions the T-cell infiltration of the brain tissue is dominated by CD8^+^ T-cells and these cells show preferential clonal expansion [4, 15]. In addition, CD20^+^ B-cells and plasma cells are present in variable numbers [12, 16]. Most importantly, therapies, which selectively target CD4^+^ T-cell responses, have been ineffective (anti CD4: [55]; Ustekinumab: [45]), while therapies which target T-cell and B-cell infiltration into the CNS (Fingolimod: [10], Alemtuzumab: [53], Natalizumab: [35]) or which selectively eliminate circulating B-cells (Rituximab: [23]) are effective.

Regarding mechanisms of demyelination we found immunoglobulin (mainly IgM) and C9neo deposition at sites of active myelin destruction and tissue damage, consisting with a pattern II demyelination according to Lucchinetti et al. [36]. The presence of lipid-specific IgM in the cerebrospinal fluid has been identified as a negative prognostic marker in MS patients [51] and co-localization of IgM with activated complement on axons and oligodendrocytes has been shown before in MS [43]. Prominent somatic hyper-mutation of IgM chains within the cerebrospinal fluid of MS patients suggests antigen specific clonal expansion of IgM producing B-cells [5].

In contrast, we did not find any indications for a pattern III type of demyelination in active lesions. Pattern III demyelination is mainly associated with massive oxidative and mitochondrial injury [1, 14, 37] and in line with this observation, oxidative injury, reflected by the accumulation of oxidized lipids in myelin and oligodendrocytes in active white matter lesions was not seen in our case. We found, however, oxidative injury in neurons and glia in demyelinating lesions in the basal ganglia, associated with high tissue iron content and extracellular iron liberation at sites of active demyelination. These data indicate that iron accumulation in the aging human brain and its liberation from myelin and oligodendrocyte stores may amplify oxidative injury in inflammatory demyelinating lesions [22].

## Conclusion

In conclusion, our study shows that a disease, which fulfills all pathological criteria for a pathological diagnosis of MS, can be induced by direct auto-immunization of humans with brain tissue. However, we also show that the detailed immunopathology of demyelinating HAE is different in many respects from that seen in classical EAE models in primates and rodents, that CD8^+^ T-cells, CD20^+^ B-cells and IgM producing plasma cells may play a decisive role in the induction of the disease and that this may be similar in MS patients. A major limitation of our current study is that it is restricted to the analysis of a single case. A basically similar pathology of inflammatory demyelination has previously been observed in a series of patients treated with the rabies semple vaccine [54]. We tried to retrieve archival autopsy material from these cases for detailed immunopathological analysis, but unfortunately we were informed that the respective material is no longer available. Most importantly, however, autoimmune encephalomyelitis in humans is a self-limiting disease. When patients survive the neuroparalytic disease after terminating rabies vaccination the patients recover without evidence for chronic (progressive) disease [50]. What drives chronic and progressive disease in multiple sclerosis remains unresolved.
